# Pharyngeal oxygenation during apnoea following conventional pre-oxygenation and high-flow nasal oxygenation

**DOI:** 10.1186/cc14280

**Published:** 2015-03-16

**Authors:** D Stolady, M Mariyaselvam, H Young, E Fawzy, M Blunt, P Young

**Affiliations:** 1 Queen Elizabeth Hospital, King's Lynn, UK

## Introduction

We hypothesised that pharyngeal oxygen concentrations would be maintained higher and for longer with transnasal humidified rapid insufflation ventilatory exchange (THRIVE) than conventional bag-mask pre-oxygenation (CPO). CPO requires the mask to be removed during laryngoscopy; this means that air may enter the mouth so subsequent apnoeic oxygenation will be less effective. Oral suctioning could exacerbate this process. However, if high pharyngeal oxygen concentrations and an open airway are maintained, apnoeic oxygenation could be substantially improved. Methods used have included NO-DESAT [1] and recently THRIVE [2], which has been shown to extend apnoea times for up to 1 hour.

## Methods

A volunteer with a nasopharyngeal sampling catheter underwent simulated emergency airway management (EAM), using both CPO and THRIVE, with and without suction. Following 3 minutes of pre-oxygenation with CPO (FiO_2_ = 1, FEO_2_ >0.8) or THRIVE (60 l/minute; Optiflow, Fisher and Paykel), EAM was simulated by voluntary apnoea and pharyngoscopy with the laryngoscope blade tip placed 2 cm from the posterior pharyngeal wall. Capnography at the laryngoscope tip confirmed apnoea. Pharyngeal gas samples (20 ml) were collected during apnoea, and after 5 seconds of oropharyngeal suctioning. Pre-oxygenation was repeated between sampling. Samples (*n *= 100) were analysed using calibrated fuel cells.

## Results

**Figure 1:**
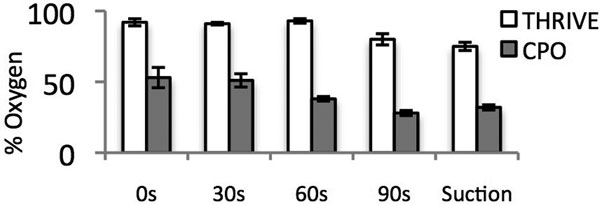
**Pharyngeal oxygen concentration**.

Pharyngeal oxygen concentrations (mean and SEM) are shown in Figure 1 (all points are significant *P *< 0.05).

## Conclusion

Pharyngeal oxygen concentration rapidly falls following CPO. This may be detrimental for apnoeic oxygenation during conventional laryngoscopy. Conversely, THRIVE maintains high pharyngeal oxygen concentrations over time. Suction has an immediate negative effect on pharyngeal oxygen concentration that is attenuated by THRIVE. Assessment of NO-DESAT (15 l/minute) was abandoned due to discomfort.
